# Upregulated SOX9 expression indicates worse prognosis in solid tumors: a systematic review and meta-analysis

**DOI:** 10.18632/oncotarget.22635

**Published:** 2017-11-06

**Authors:** Haihua Ruan, Shuangyan Hu, Hongyu Zhang, Gang Du, Xiaoting Li, Xiaobo Li, Xichuan Li

**Affiliations:** ^1^ Tianjin Key Laboratory of Food Science and Biotechnology, College of Biotechnology and Food Science, Tianjin University of Commerce, Tianjin, China; ^2^ Department of Immunology, Tianjin Medical University, Tianjin, China

**Keywords:** solid tumors, SOX9, prognosis, meta-analysis

## Abstract

It was recently reported that increased SOX9 expression drives tumor growth and promotes cancer invasion during human tumorigenicity and metastasis. However, the prognostic value of SOX9 for the survival of patients with solid tumors remains controversial. The present meta-analysis was thus performed to highlight the link between dysregulated SOX9 expression and prognosis in cancer patients. A systematic literature search was conducted using the electronic databases PubMed, Web of Science and Embase to identify eligible studies. A random-effects meta-analytical model was employed to correlate SOX9 expression with overall survival (OS), disease-free survival (DFS) and clinicopathological features. In total, 17 studies with 3307 patients were eligible for the final analysis. Combined hazard ratios (HRs) and 95% confidence intervals (CIs) suggested that high SOX9 expression has an unfavourable impact on OS (HR = 1.66, 95% CI 1.36–2.02, *P* < 0.001) and DFS (HR = 3.54, 95% CI 2.29–5.47, *P* = 0.008) in multivariate analysis. Additionally, the pooled odds ratios (ORs) indicated that SOX9 over-expression is associated with large tumor size, lymph node metastasis, distant metastasis and a higher clinical stage. Overall, these results indicated that SOX9 over-expression in patients with solid tumors might be related to poor prognosis and could serve as a potential predictive marker of poor clinicopathological prognosis factor.

## INTRODUCTION

SOX9 is a member of SOX [SRY (sex determining region Y)-related high mobility group (HMG) box] family and serves as a transcription factor that plays a central role in the development and differentiation of multiple cell lineages [1]. Discovery of SOX9 began with its function underlying campomelic dysplasia (CD), a rare genetic disorder characterized by bowing of the long bones [1]. In the past decade, the knowledge of SOX9 has developed rapidly. SOX9 plays a versatile role in chondrogenesis and skeletal development, in male gonad genesis, in differentiation of multiple organs, in ectoderm development, and in various solid tumors [2–7].

Increased SOX9 expression drives prostate cancer (PCa) tumor growth and angiogenesis and promotes prostate cancer invasion by reactivating the WNT/β-catenin signaling that mediates ductal morphogenesis in fetal prostate [8]. SOX9 overexpression significantly induces the proliferation and tumorigenicity of human esophageal squamous cell cancer (ESCC) cells by increasing the expression of phosphorylated Akt and its downstream targets such as phosphorylated forkhead box O (FOXO) 1 and phosphorylated FOXO3, two members of FOXO family of transcription factors [9]. Aberrant SOX9 expression contributes to the development of gastric cancer by inactivation of GKN1 as an early event [10]. Conversely, knockdown of SOX9 suppresses chondrosarcoma growth and migration [11], and induces apoptosis, cell cycle arrest as well as decreased expression of cancer stem cell markers [12–14]. Therefore, inhibited tumor growth and invasion by SOX9 knockdown shed light on regarding SOX9 as a therapeutic target for cancer. A plenty of studies investigated the correlation between SOX9 expression and prognosis in cancer patients, and demonstrated that upregulated expression of SOX9 in malignant tumors was correlated with poor prognosis in patients with different types of solid tumors such as chordoma [13], osteosarcoma [14–16], colorectal carcinoma [17, 18], esophageal squamous cell carcinoma [10, 19], breast cancer [20–23], hepatocellular carcinoma (HCC) [24, 25], glioma [26], chondrosarcoma [27], gastric cancer [28–30], melanoma [31], pancreatic ductal adenocarcinoma (PDAC) [32], ovarian cancer (OC) [33], prostate cancer [34, 35] and non-small cell lung cancer (NSCLC) [36]. However, some other studies revealed that overexpression of SOX9 was not significantly associated with prognosis of some patients with gastric cancer [9] and with breast cancer when looking at overall or 5-year survival [37]. Taken together, the exact clinical and prognostic merit of SOX9 overexpression in various solid tumors remains unclear. Moreover, most of these studies included only a limited number of patients, and the results of each individual study were not conclusive.

In this study, we herein issued a comprehensive meta-analysis to appraise the prognostic significance of SOX9 overexpression in solid human tumors, and illustrate the clinical value of SOX9 as a prognostic indicator and potential therapeutic target for malignant tumor patients.

## RESULTS

### Study search information

**Figure 1:**
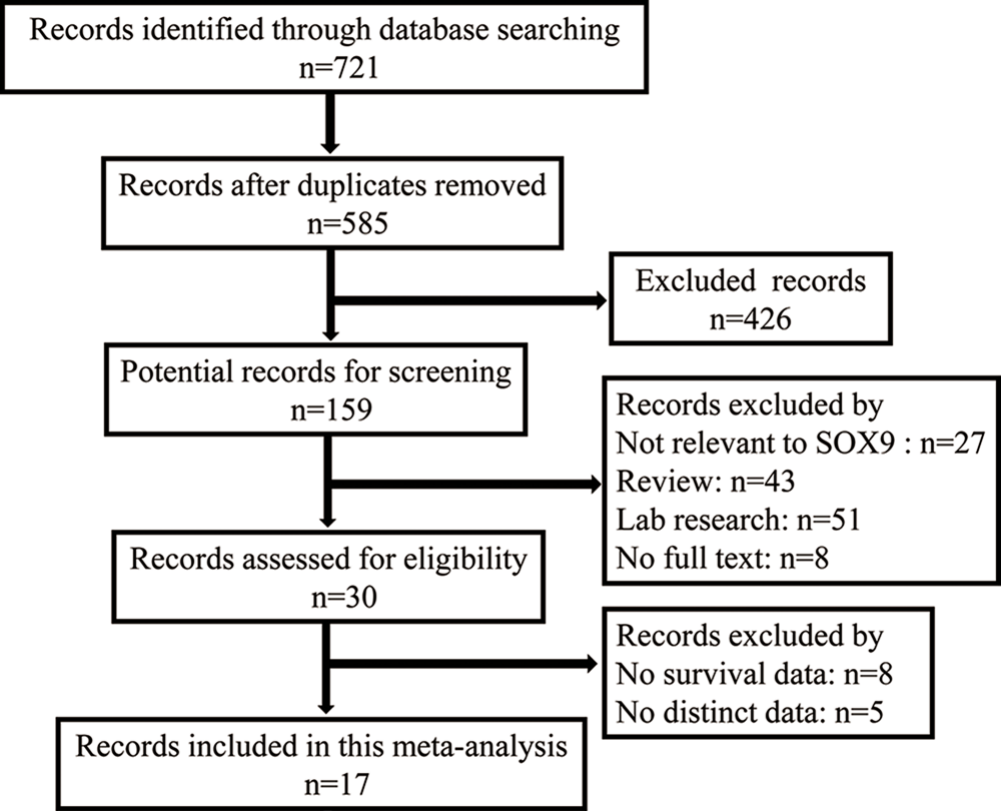
Flow diagram of the selection of eligible studies

The initial search identified 721 publications, of which, 30 studies were of acceptable relevance. However, eight of these studies were excluded because the absence of survival data, and five were excluded because of the absence of information about distinct data. Ultimately, 17 studies met the eligibility criteria and were included in the current meta-analysis (Figure 1).

### Description of the studies

The main characteristics of the 17 identified studies were presented in Table 1. In total, 3307 patients from five regions (China, Korea, United States of America, Australia and Japan) with 11 distinct cancers, chordoma [13], osteosarcoma [14, 16], esophageal cancer [9, 19], hepatocellular carcinoma [24, 38], intrahepatic cholangiocarcinoma [39], pancreatic ductal adenocarcinoma [32], prostate cancer [34, 35], thyroid carcinoma [40], colorectal cancer [18, 41], gastric cancers [10, 42], non-small cell lung cancer [36] were included in these studies.

**Table 1:** Main characteristics of studies exploring the relationship between SOX9 expression and tumor prognosis

Author	Year	Region	Cancer Type	Stage / Grade	No. of Patients	Follow-up Time Median (range)	Detection Method	Cut-off	NOS Score	Outcomes
Chen H [13]	2017	USA	Chordoma	I-III	50	4-250 m	IHC(Santa Cruz)	PS > 2	5	OS, DFS
Qi J [14]	2017	China	Osteosarcoma	I-III	97	10-72 m	IHC(Santa Cruz)	IRS > 5	6	OS
Yang Z [19]	2016	Korea	Esophageal cancer	I-V	127	1-120 m	IHC(Abnova)	NR	6	OS, DFS
Liu C [24]	2016	China	Hepatocellular Carcinoma	I-III	148	1-80 m	IHC(Millipore)	PS > 2	6	OS
Hong Y [9]	2015	China	Esophageal cancer	I-V	155	1-100 m	IHC(Abcam)	IRS > 6	7	OS
Matsushima H [39]	2015	Japan	Intrahepatic cholangiocarcinoma	I-V	43	1-150 m	IHC(Abcam)	NR	5	OS
Xia S [32]	2015	China	Pancreatic ductal adenocarcinoma	I-V	88	1-60 m	IHC(Millipore)	IRS > 6	6	OS
Qin GQ [34]	2014	China	Prostate cancer	T2A	98	1-140 m	IHC(Santa Cruz)	PS > 1	7	OS, DFS
Zhu H [16]	2013	China	Osteosarcoma	II-III	166	10–152 m	IHC(Santa Cruz)	IRS > 5	6	OS, DFS
Yun JY [40]	2013	Korea	Thyroid carcinoma	I-V	158	47.5 m for median	IHC(Abnova)	PS > 1	7	OS
Candy P [41]	2013	Australia	Colorectal cancer	I-III	1056	69.7 m for median	IHC(Santa Cruz)	> 50%	8	OS
Choi YJ [10]	2013	Korea	Gastric cancers	NR	185	1-60 m	IHC(Millipore)	> 30%	7	OS
Zhong WD [35]	2012	China	Prostate cancer	T2A	147	3-12 y	IHC(Santa Cruz)	IRS > 4	6	DFS
Guo X [38]	2012	China	Hepatocellular Carcinoma	I-V	130	8.6 year for median	IHC(Santa Cruz)	IRS > 5	7	OS, DFS
Zhou CH [36]	2012	China	Non-small cell lung cancer	I-V	89	1-60 m	IHC(Millipore)	IRS > 6	6	OS
Sun M [42]	2012	China	Gastric cancer	NR	382	1-3000 d	IHC(Millipore)	IRS > 5	8	OS
Lü B [18]	2008	China	Colorectal Cancer	I-V	188	1-12.5 y	IHC(Santa Cruz)	PS > 2	7	OS

NR: Not Reported; y: year; m: month; d: day; OS: Overall Survival; DFS: Disease-Free Survival. PS: Percentage Score; IRS: Immunoreactive Score.

### Correlations between SOX9 expression and OS

**Figure 2:**
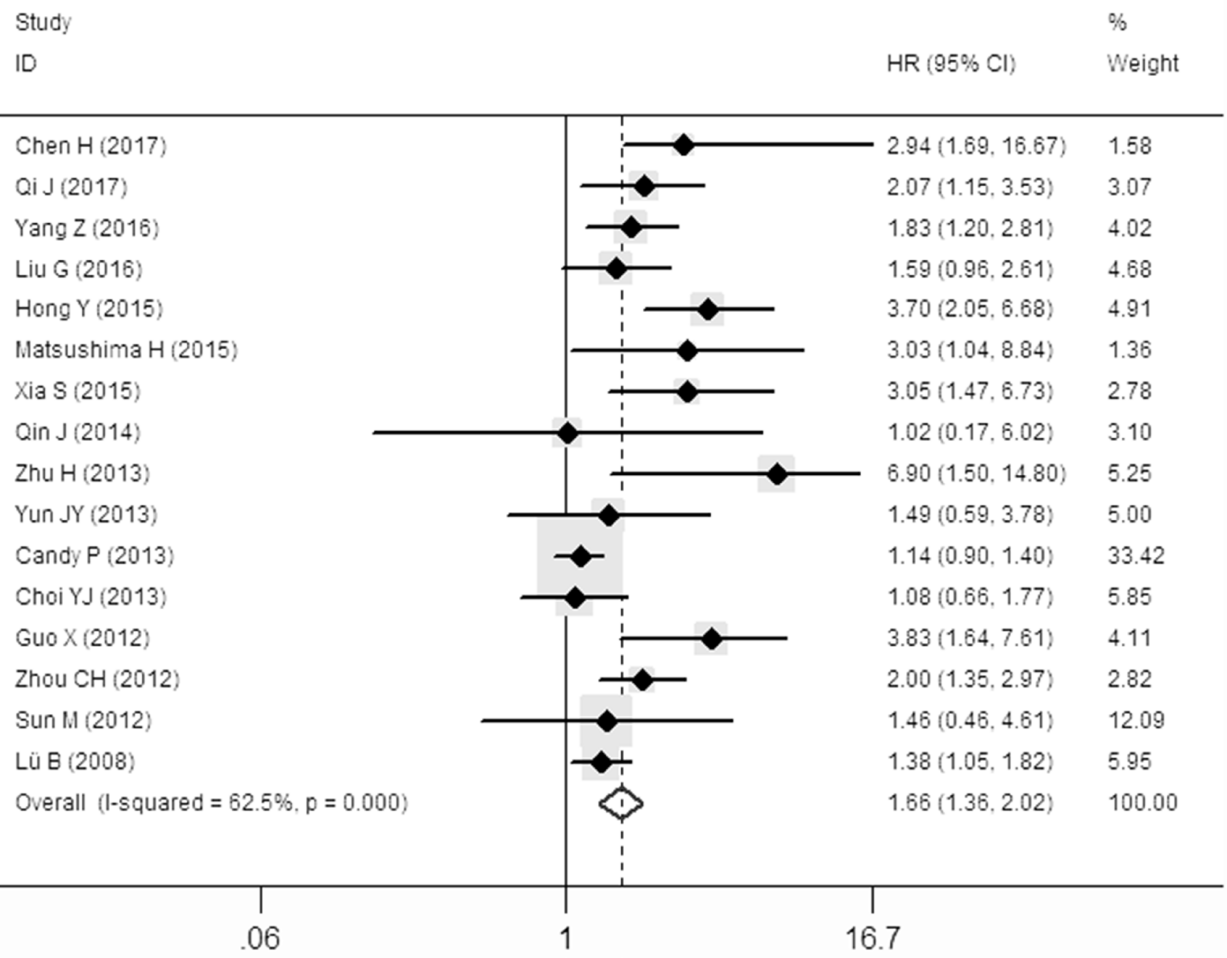
Forest plot describing the association between over-expressed SOX9 and OS

The pooled hazard ratio (HR) revealed that over-expressed SOX9 was significantly associated with poor overall survival (OS) for cancer victims in multivariate analysis (HR: 1.66, 95% CI: 1.36–2.02; Figure 2). However, a significant heterogeneity (I^2^ = 62.5%, *P* = 0.001) was observed when using a random-effects model to analyze the pooled HR of the OSs.

To minimize heterogeneity, the subgroup analyses were performed according to the ethnics (Asian or not), case number (≥ 100 or not), NOS score (≥ 7 or not), follow-up time (≥ 120 m or not), antibody (various company), cut-off value (various scoring criteria). The pooled HRs and heterogeneities according to all these factors were presented in Table 2. Unfortunately, all these subgroup analyses demonstrated that there were no significant lower I^2^ value when the *P* < 0.05. Therefore, subgroup analysis were failed to find the origin of high heterogeneity.

**Table 2:** Associations between SOX9 expression and OS stratified according to the ethnics, case number, NOS score, follow-up time, antibody and cut-off value

Categories	Subgroups	Ref	HR (95% CI)	Heterogeneity test (I^2^, *P*-value)
Ethnics	Asian Not Asian	[9, 10, 14, 16, 18, 19, 24, 32, 34–36, 38–40, 42]	1.98 (1.50–2.62)	53.8%, 0.009
			1.19 (0.96–1.48)	60.7%, 0.111
Case Number	≥ 100	[13,41]	1.60 (1.29–1.99)	71.8%, 0.000
		[9, 10, 18, 19, 24, 32, 34, 35, 38, 39, 42]		
	< 100	[13, 14, 16, 36,40, 41]	2.05 (1.30–3.23)	0.0%, 0.770
NOS Score	≥ 7	[9, 10, 18, 34, 38, 40–42]	1.41 (1.10–1.79)	67.5%, 0.003
	< 7	[13, 14, 16, 19, 24, 32, 35, 36, 39]	2.69 (1.99–3.62)	46.4%, 0.071
Follow-up Time	≥ 120 m	[13, 16, 18, 19, 34, 35, 39]	2.26 (1.46–3.50)	78.0%, 0.001
	< 120 m	[9, 10, 14, 24, 32, 36, 38, 40–42]	1.53 (1.23–1.91)	67.1%, 0.001
Antibody	Santa Cruz	[13, 14, 16, 18, 34, 35, 38, 41]	1.58 (1.28–1.95)	74.0%, 0.001
	Millipore	[10, 24, 32, 36, 42]	1.54 (0.92–2.59)	41.1%, 0.147
	Abcam	[9, 39]	3.54 (2.11–5.94)	0.0%, 0.749
	Abnova	[19, 40]	1.63 (0.94–2.83)	0.0%, 0.573
Cut-off Value	IRS	[9, 14, 16, 32, 35, 36, 38, 42]	2.64 (1.67–4.17)	30.3%, 0.197
	PS	[13, 18, 24, 34, 40]	1.47 (0.99–2.18)	0.0%, 0.760
	Percentage	[10, 41]	1.13 (0.92–1.38)	0.0%, 0.844
	NR	[19, 39]	2.08 (1.37–3.16)	0.0%, 0.366

m: month; PS: Percentage Score; IRS: Immunoreactive Score; NR: Not Reported.

### Correlations between SOX9 expression and DFS

**Figure 3:**
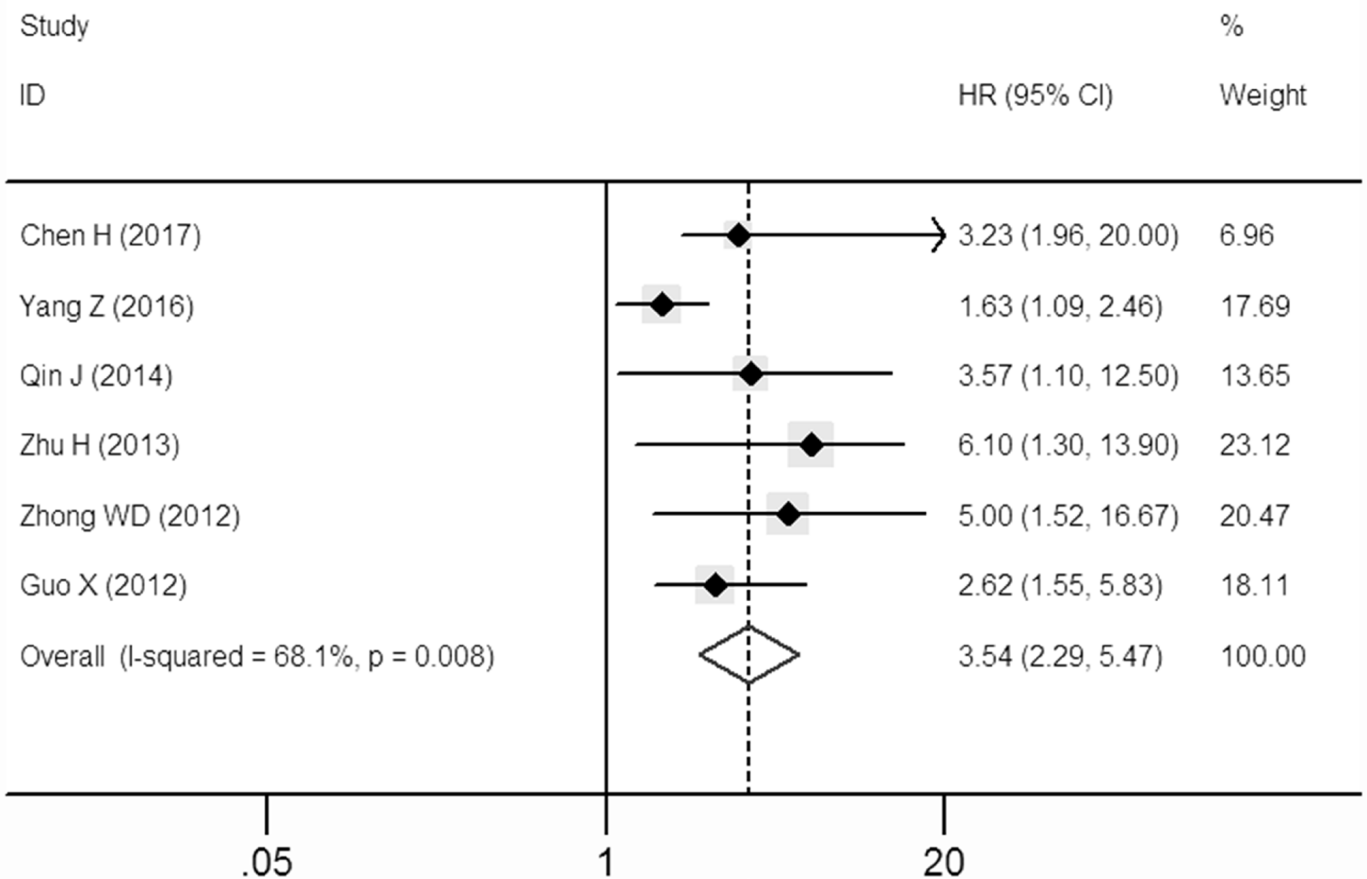
Forest plot describing the association between over-expressed SOX9 and DFS

A significant correlation between over-expressed SOX9 and disease-free survival (DFS) was also observed in the patients with solid tumors in multivariate analysis (HR: 3.54, 95% CI: 2.29–5.47; Figure 3) in the random-effects model with a significant heterogeneity (I^2^ = 68.1%, *P* = 0.008).

### Correlations between SOX9 expression and clinicopathological parameters

The clinical and pathological parameters collected from the eligible studies were presented in Supplementary Table 1. Meanwhile, pooled results of the correlations were identified between over-expressed SOX9 and clinicopathological features of patients with solid tumors. No significant correlations between over-expressed SOX9 with gender and tumor differentiation were observed. However, the expression of SOX9 was positively associated with tumor size (OR: 1.58, 95% CI: 1.31–1.91), lymph node metastasis (OR: 1.61, 95% CI: 1.30–1.99), distant metastasis (OR: 1.53, 95% CI: 1.25–1.87) and a higher clinical stage (OR: 1.68, 95% CI: 1.33–2.12) in the random-effects model with significant heterogeneities (see Table 3 and Supplementary Figure 1).

**Table 3:** Meta-analysis results of the associations of increased SOX9 expression with clinicopathological parameters

Clinicopathological parameter	Ref	Overall OR (95% CI)	Heterogeneity test (I^2^, *P*-value)
Gender (male vs female)	[9, 14, 16, 18, 19, 32, 38, 39]	0.99 (0.85–1.15)	0.0%, 0.439
Tumor Differentiation (poor VS well)	[9, 18, 19, 32, 38, 39]	1.13 (0.93–1.39)	59.2%, 0.031
Tumor Size (T3-4 vs T1-2)	[9, 14, 16, 18, 19, 32, 38, 39]	1.58 (1.31–1.91)	81.3%, 0.000
Lymph Node Metastasis (yes vs no)	[9, 18, 19, 32, 39]	1.61 (1.30–1.99)	84.9%, 0.000
Distant Metastasis (yes vs no)	[9, 14, 16, 19, 32, 39]	1.53 (1.25–1.87)	27.3%, 0.230
Clinical Stage (III-IV vs I-II)	[9, 14, 16, 18, 19, 32, 39]	1.68 (1.33–2.12)	90.4%, 0.000

### Assessment of heterogeneity and sensitivity

**Figure 4:**
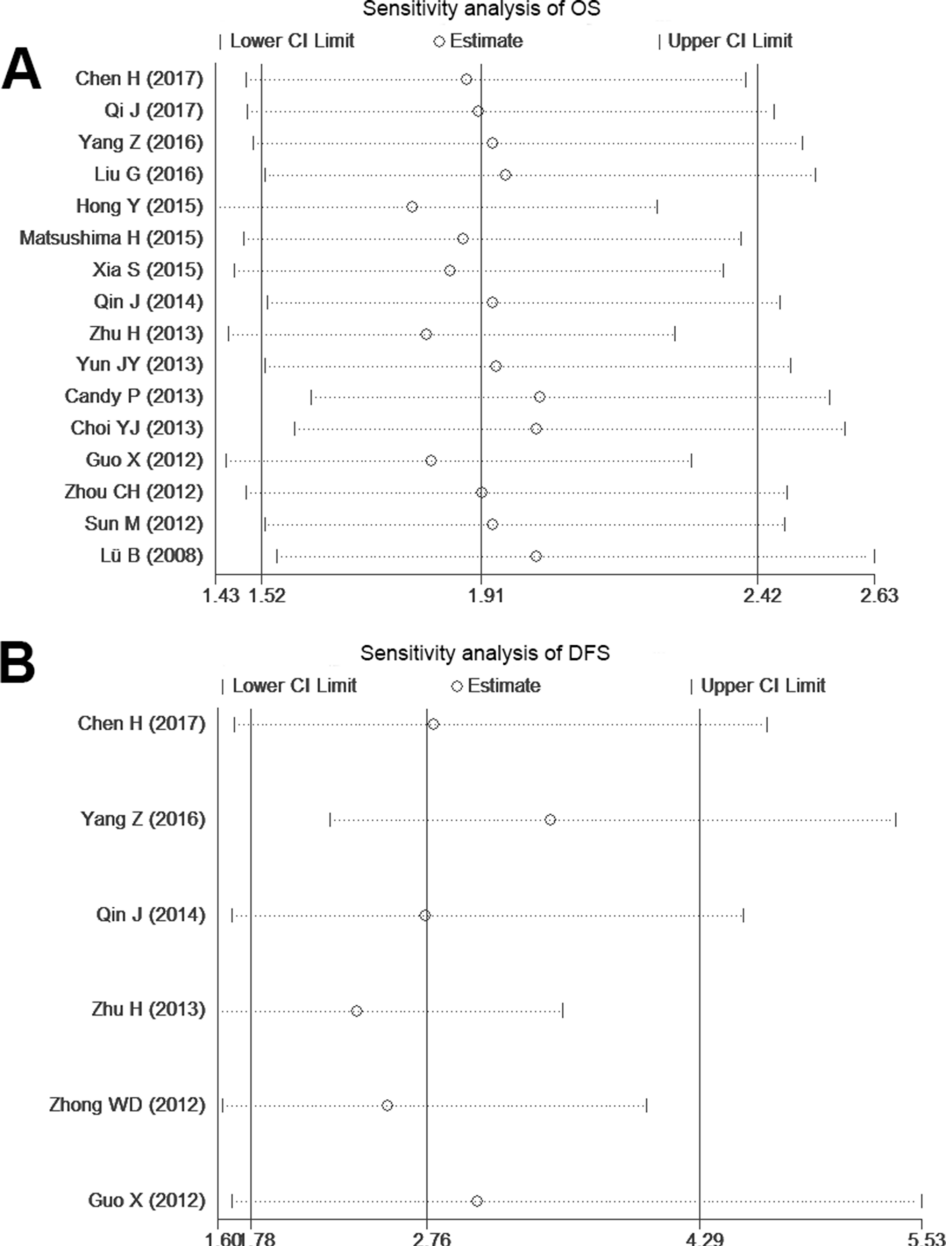
Sensitivity analysis of the OS and DFS in the meta-analysis

There was significant heterogeneities (I^2^ > 50%) between studies in OS and DFS analyses. So the random-effect model was therefore adopted in these studies. A meta-regression analysis with published country, case number (≥ 100 or not), antibody (used for different companies) and cut-off value (scores or not) as covariates was conducted. All covariates were fit into the meta-regression model one at a time to identify potential sources of heterogeneity. In terms of OS and DFS, none of these covariates were verified as a significant source of heterogeneity (Table 4). Also, by successively omitting each study from the aggregated survival meta-analyses, sensitivity analysis was performed to evaluate the influence of each individual study on the pooled HR of OS and DFS (Figure 4). The results revealed that the pooled estimates of the effect of over-expressed SOX9 on the OS and DFS of patients with solid tumors did not vary substantially with the exclusion of any individual study, which implies that the results of this meta-analysis are stable.

**Table 4:** Results of meta−regression analysis exploring source of heterogeneity with OS and DFS

Covariates	OS			DFS		
	Coef.	S.E.	*P* value	Coef.	S.E.	*P* value
Country	−0.129	.083	0.144	−0.310	0.328	0.399
Case Number	0.263	0.261	0.331	0.227	0.576	0.713
Antibody	0.033	0.122	0.792	−0.250	0.103	0.071
Cut-off value	−0.065	0.121	0.599	0.310	0.123	0.065

Coef.: Coefficient; S.E.: Standard Error;

### Publication bias

**Figure 5:**
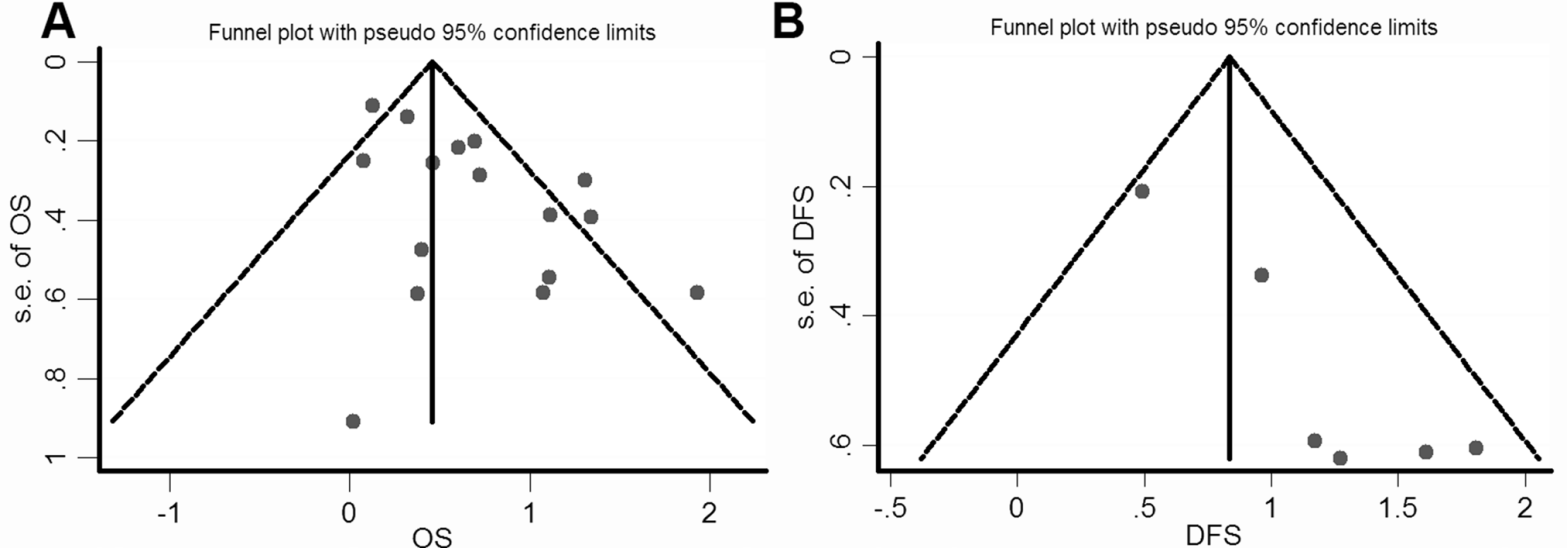
Funnel plot for the assessment of potential publication bias regarding OS and DFS in the meta-analysis

We constructed funnel plots and performed Begg's test to assess publication bias. As a result, the shape of the funnel plot for the OS, DFS and clinicopathological parameters seemed symmetrical in the multivariate analysis method (Figure 5 and Supplementary Figure 2). The Begg's and Egger's tests revealed non-significant values (*P* = 0.322 and 0.08, respectively).

## DISCUSSION

The transcription factor SOX9 is a member of SOX family proteins which contain a highly conserved HMG domain that was first identified in Sry, an essential factor involved in mammalian male sex determination [43]. In general, proteins containing a domain with 50% or higher amino acid similarity to the HMG are referred to as SOX proteins. Around 20 SOX proteins have been confirmed in mice and humans, and are grouped A through H based on the structural homology outside of their HMG boxes. SOX9 belongs to SoxE proteins [44] and exerts its function in sex determination, cell differentiation during embryonic development, and cell maintenance and specification during adult life of mice and human [2].

Since the first record on the analysis of SOX9 expression in human cancer published in 1997 [45], more than hundreds studies have explored the role of SOX9 expression in tumors in larger patient groups. SOX9 is over-expressed in various human malignancies and growing evidence demonstrates its association with human solid tumor growth [9, 10]. Conversely, knockdown of SOX9 provides inhibition of chondrosarcoma growth and migration, and induces apoptosis and cell cycle arrest [26]. The meta-analysis presented herein is the first comprehensive description of all reported survival data from 3307 solid tumor patients from 17 eligible studies, which met the inclusion criteria, investigating the impact of SOX9 expression in human tumors on prognosis. For all studies, SOX9 expression was detected by IHC. By meta-analysis of the 17 studies, we identified the pool HRs which indicated that SOX9 was a factor in poor prognosis in various cancers. Because there is no significant heterogeneity among our included studies, so we did not perform further subgroup analyses.

For the reasons of SOX9 overexpression correlated with poor prognosis in various solid tumors, we summarized as follows: i) Downregulated expression of E-cadherin and increased expression of βcatenin, which are key factors for epithelial–mesenchymal transition (EMT) in gastric cancers, by SOX9 overexpression. Aberrant SOX9 expression inactivates the activity of gastrokine 1 (GKN1) [46]. Inactivation of GKN1 downregulates expression of E-cadherin and increases expression of βcatenin in gastric cancers [46]. Besides that, SOX9 activates TGFβ/Smad signaling, activation of this signaling pathway upregulates snail expression, which in turn triggers EMT, resulting in down-regulation of E-cadherin and increased expression of βcatenin [47]. Overexpression of βcatenin leads to the induction of EMT in gastric cancers and partially restores the colonyforming potential in squamous cell cancer (SCC) development [48]. ii) SOX9 is important in maintaining the properties of cancer stem cell (CSC) in various tumors. The hedgehog (Hh) pathway is involved in CSC maintenance in various tumors [49]. Glioma-associated oncogene homolog 1 (Gli1) is a key mediator of the Hh pathway; involved in CSC maintenance [49]. Gli1 expression is correlated with the expression of stemness genes, SOX9, and cell cycle regulators such as p21, cyclin D1, cyclin E1, and NF-κB, which are strongly linked to worse clinical outcome and independent poor prognostic factors in overall survival and disease-free survival in ESCC [19]. iii) Enhanced transcription of SOX9 responsive genes during tumorgenecity. SOX9 is showed to bind to 4293 genes in common between the mouse and bovine genomes [50]. Most of these genes are already known to be involved in sex determination. Moreover, transcriptomic (RNA-seq) analysis of foetal testes from SOX9 knockout mice showed that SOX9 not only regulates transcription of its target genes directly, but also influences their RNA splicing [50]. Thus, in great possibility, the overexpressed SOX9 might results in disordered gene expression in tumorgenecity. For example, SOX9 transcriptionally activated FOXK2, which belongs to the fork head DNA binding protein family, has been shown to play a critical role in tumorigenesis, high expression of FOXK2 is significantly correlated with poor survival of colorectal cancer [51]. iv) SOX9 promotes osteosarcoma (OS) cell growth by inhibiting the promoter activity of the CLDN8 gene and down-regulating CLDN8 expression, which functions as an oncogenic factor and was up-regulated in OS cells [14]; Overexpression of SOX9 in adult mouse prostate epithelia induces an early high-grade prostate intraepithelial neoplasia (PIN) lesion, indicating that SOX9 augments the loss of PTEN, which is a factor vital for tumor formation [52].

Additionally, no publication bias was observed. Our meta-analysis results involve several important implications. First, it shows that over-expressed SOX9 was positively related to poor OS and DFS in solid tumor patients. Second, pooled results of the correlations were identified between over-expressed SOX9 and clinicopathological features of patients with solid tumors, indicating that SOX9 may serve as a promising therapeutic target. Third, our results showed the expression of SOX9 was positively associated with lymph node metastasis, large tumor size, distant metastasis and a higher clinical stage. We can explain this result by SOX9's ability to enhance prostate cancer (PCa) tumor growth, promote tumor cell proliferation, invasion and metastasis [31]. Because of its involvement in these processes, SOX9 is likely to be causally involved in tumor progression and, consequently, increased levels of SOX9 would be expected to indicate a poor prognosis. Finally, it highlights the potential clinical application of SOX9 as a valuable prognostic biomarker.

This meta-analysis was properly performed, however, further analysis with several limitations would be considered in the future. Firstly, need more trials to analysis; second, some of the survival data were extracted from Kaplan-Meier curves and might be less reliable than a direct analysis of variance; third, we need to search more non-English publications. In addition, the possible existence of unpublished studies could also result in potential publication bias. In general, concerning these limitations mentioned above, a larger cohort sample size, adjusted individual data and a unified detection method are required to achieve a more persuasive conclusion.

In conclusion, our meta-analysis demonstrated that over-expressed SOX9, as evaluated by IHC, is positively related to poor OS and DFS in human solid tumor patients. Over-expressed SOX9 could be served as a potential biomarker for unfavorable clinicopathological prognostic factors in patients with various solid tumors, suggesting that directly targeting SOX9 could be promising therapeutic approaches for solid malignancies.

## MATERIALS AND METHODS

### Literature search strategy

This systematic review and meta-analysis is reported in accordance with the Preferred Reporting Items for Systematic Review and Meta-Analysis (PRISMA) statement [53]. We performed a thorough search of PubMed, Embase and Web of Science databases for studies measuring expression of SOX9 and survival in patients with solid tumors from 1997 to August 2017.

The search terms included the following key words in various combinations: SOX9, prognosis, prognostic, survival, and overall survival. The hits were restricted to human studies of solid tumors and those published in English. The references list of review and bibliographies were further sifted to identify additional potentially relevant studies to avoid omission due to the electronic search approach.

### Study inclusion and exclusion criteria

The collected studies included in this meta-analysis had to meet the following criteria: (1) a pathological diagnosis of cancer was made; (2) SOX9 expression in patients with any type of tumor was measured via immunohistochemistry; (3) associations of SOX9 expression with OS, DFS or clinicopathological features were described; (4) HRs and 95% confidence intervals (CIs) were reported or could be calculated (based on the information in the paper); and (5) when the same author reported repeated results from the same population, the most complete report was included. The exclusion criteria for this meta-analysis were as follows: (1) unpublished papers; (2) laboratory articles, reviews and letters; (3) non-English language articles; (4) overlapping articles or ones with duplicate data; (5) articles with only animal experiments; (6) studies without information about survival curves; and (7) SOX9 expression in patients with any type of tumor was analyzed only using RT-PCR method.

### Data extraction and quality assessment

All data were extracted independently by two investigators (Haihua Ruan and Xichuan Li). For each eligible study, the following characteristics were extracted: first author's name, publication year, region, type of cancer, number of patients, patients’ ages, follow-up times, detection methods, cut-off values, survival data (including OS and DFS) and clinicopathological parameters, such as gender, tumor differentiation, tumor size, lymph node metastasis, distant metastasis and clinical stage. For studies that presented only Kaplan-Meier curves was used to extract the survival data. The cut-off values of SOX9 expression were differently indicated among the included studies. Briefly, the percentage scoring (PS) of immunoreactive tumor cells was calculated as follows: 0 (0 %), 1 (1–25 %), 2 (26–50 %), 3 (51–75 %) and 4 (76–100 %). The staining intensity was visually scored and stratified as follows: 0 (negative); 1 (weak); 2 (moderate); and 3 (strong). The immunoreactivity score (IRS) was obtained in some studies by multiplying the percentage and the intensity score.

### Statistical analysis

This meta-analysis was performed using Stata 12.0 (Stata Corporation, College Station, TX, USA) software. Pooled estimates of HRs and their 95% CIs were used to estimate the association between SOX9 expression and patients’ survival. The chisquared test (Cochrane’ s Q test) and I-squared statistical test were used to analyze the heterogeneity between studies. When the result of a Q-test (I^2^ > 50% or *P* < 0.05) indicated heterogeneity, the random-effects model was used for the meta-analysis. Otherwise, a fixed-effects model was used. HR with its 95% CI over 1.0 indicated poor prognosis patients with increased SOX9 expression. Funnel plots were used to graphically represent the publication bias. Begg's (rank correlation) test was adopted to confirm the publication bias. Begg's (rank correlation) and Egger's (regression asymmetry) tests were adopted to confirm the publication bias.

## SUPPLEMENTARY MATERIALS FIGURES AND TABLES


