# Supplementary material for: Upregulated SOX9 expression indicates worse prognosis in solid tumors: a systematic review and meta-analysis
Source: Oncotarget. 2017 Nov 6;8(68):113163–73. doi: 10.18632/oncotarget.22635 (PMC5762580; doi:10.18632/oncotarget.22635)
Supplement: Supplementary file 1 [file oncotarget-08-113163-s001.pdf]

Upregulated SOX9 expression indicates worse prognosis in solid tumors: a systematic review and meta-analysis

SUPPLEMENTARY MATERIALS

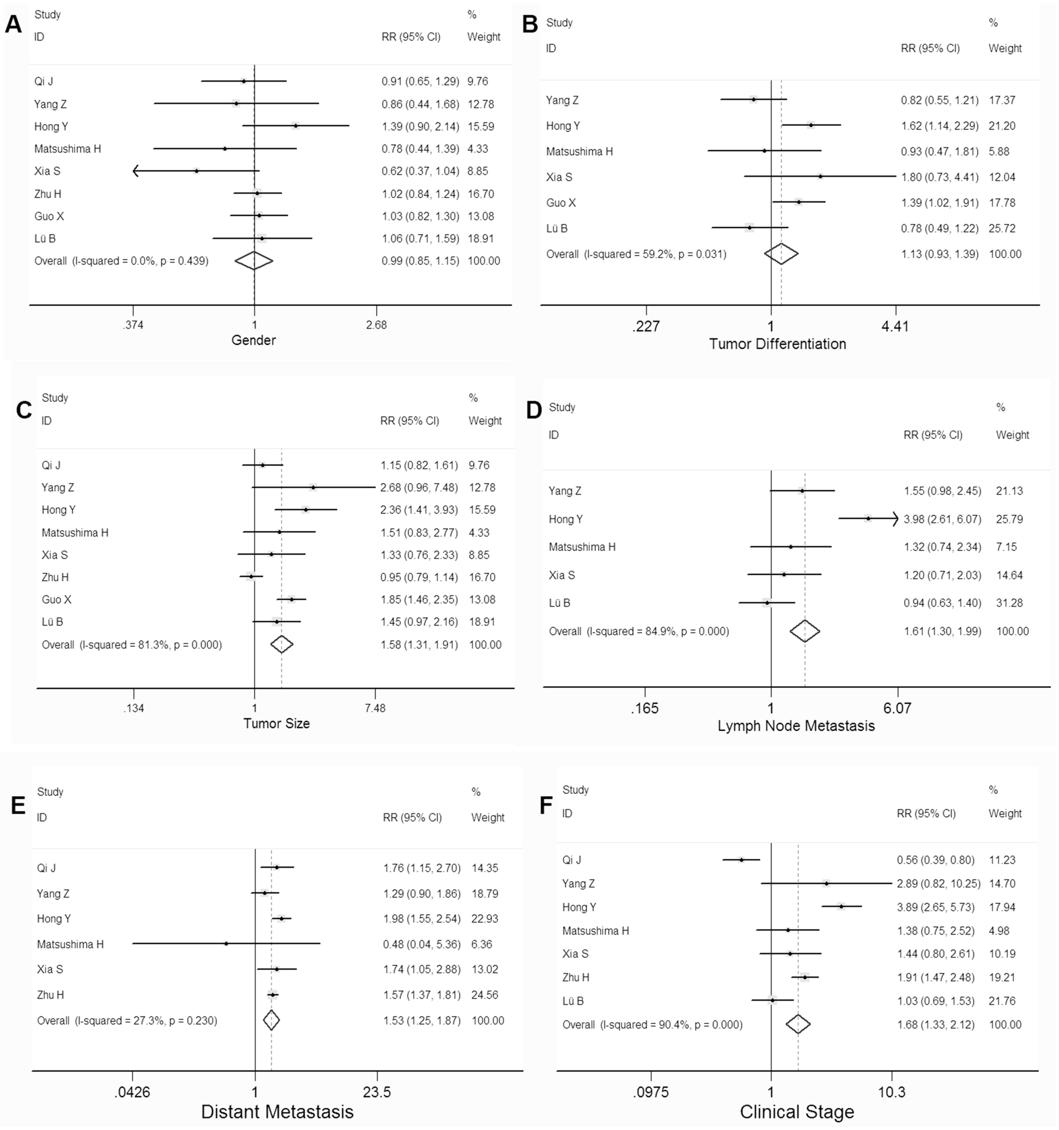

Supplementary Figure 1: Forest plot describing the association between over-expressed SOX9 and clinicopathological parameters.

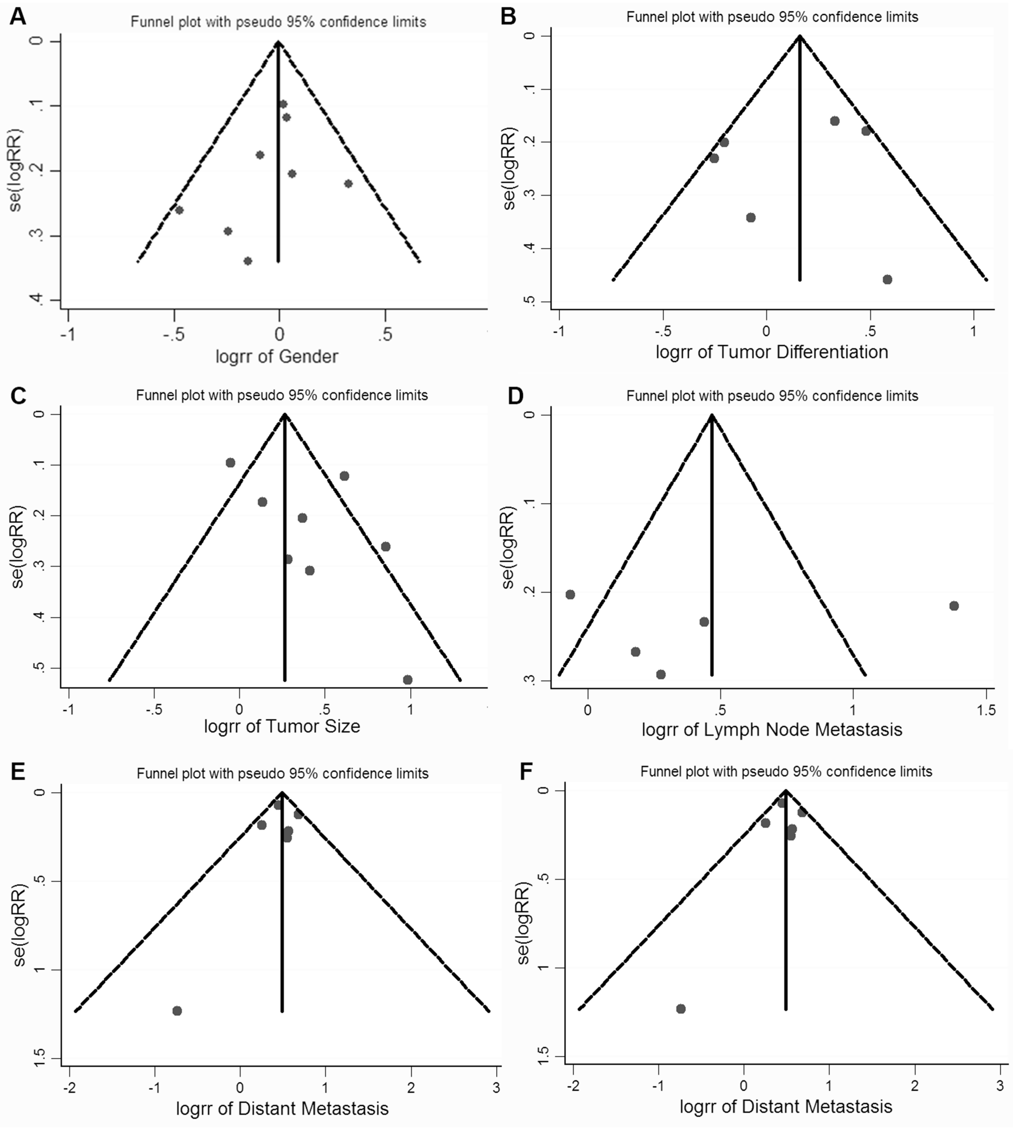

Supplementary Figure 2: Funnel plot for the assessment of potential publication bias regarding clinicopathological parameters in the meta-analysis.

Supplementary Table 1: Summarized data of clinical and pathological parameters from the eligible studies

First author	Gender				Tumor Differentiation				Tumor Size				Lymph Node Metastasis				Distant Metastasis				Clinical Stage			
	Male	Female			Poor+Moderate/ undifferentiated	Well/ differentiated		T3-4		T1-2		Yes		No		Yes		No		III-IV		I-II		
SOX9	−	+	−	+	−	+	−	+	−	+	−	+	−	+	−	+	−	+	−	+	−	+	−	
Qi J [14]	34	27	22	14	NR	NR	NR	NR	22	13	34	28	NR	NR	NR	NR	41	18	15	23	22	30	34	11
Yang Z [19]	59	61	4	3	49	54	14	10	60	52	3	12	49	39	14	25	18	12	45	52	61	55	2	9
Hong Y [9]	62	54	15	24	51	34	26	44	65	43	12	35	59	11	18	67	7	0	70	78	56	7	21	71
Matsushima H [39]	12	14	10	7	17	16	5	4	13	8	9	13	8	5	14	16	0	1	22	20	13	9	9	12
Xia S [32]	19	40	15	14	30	41	4	13	22	29	12	25	17	23	17	31	12	9	22	45	23	35	11	29
Zhu H [16]	70	26	50	20	NR	NR	NR	NR	62	26	58	20	NR	NR	NR	NR	42	0	78	46	88	10	32	36
Guo X [38]	73	23	25	9	80	19	18	13	65	2	33	30	NR	NR	NR	NR	NR	NR	NR	NR	NR	NR	NR	NR
Lü B [18]	36	67	28	57	18	45	46	79	22	28	42	96	32	65	32	59	NR	NR	NR	NR	30	56	33	64

NR: Not Reported
